# Supplementary material for: Gut Microbiota and Bile Acids Mediate the Clinical Benefits of YH1 in Male Patients with Type 2 Diabetes Mellitus: A Pilot Observational Study
Source: Pharmaceutics. 2022 Sep 2;14(9):1857. doi: 10.3390/pharmaceutics14091857 (PMC9505101; doi:10.3390/pharmaceutics14091857)
Supplement: Supplementary file 1 [file pharmaceutics-14-01857-s001.zip › pharmaceutics-1894473-supplementary.pdf]

Supplementary material – Protocol

Protocol S1. Analysis of stool and plasma bile acids as well as plasma C4.

About 50 mg of stool samples were placed in a 2-ml polypropylene tube together with six ceramic beads (3 mm; Retsch GmbH, Haan, Germany) and 500 µl of internal standard containing methanol. Stools were homogenized and centrifuged, and the supernatant will be diluted ten times in methanol:water (1:1) before analysis.

Human plasma (100 µL) was added to 300 µL of methanol for protein precipitation and centrifuged for 10 min at 12000 g at 4°C. Supernatant was injected onto the UPLC-MS/MS system for bile acids analysis.

The LC analysis was performed on a Waters ACQUITY Ultra Performance Liquid Chromatography system (Waters, USA). Chromatographic separation of plasma was carried out at 60°C on a Waters ACQUITY BEH C8 column (2.1 mm × 100 mm × 1.7 µm). The mobile phase A was consisting of 10% acetonitrile (containing 0.01% formic acid) and mobile phase B was 50% isopropanol in acetonitrile containing 0.01% formic acid. The flow rate was set at 1.3 mL/min.

Mass spectrometry was performed on a XEVO TQ-XS mass spectrometer. All bile acids were detected in the negative ionization mode. The capillary voltage was 1.5 kV and the source temperature was 150°C. The desolvation gas was set at 1000 L/h at a temperature of 600°C. The cone gas flow was 150 L/h. The multiple reactionmonitoring (MRM) transitions for analytes are shown in Table 1. System operation and data acquisition were controlled using Mass Lynx software, and targeted metabolic data were analyzed by TargetLynx (Waters, Milford, USA).

Compound Name	Abbreviation	Parent (m/z)	Daughter (m/z)	Cone (V)	Collision (V)
**Lithocholic acid**	LCA	375.25	375.25	60	32
**Chenodeoxycholic acid**	CDCA	391.25	391.25	60	16
**Deoxycholic acid**	DCA	391.25	391.25	60	16
**Ursodeoxycholic acid**	UDCA	391.25	391.25	60	16
**Cholic acid**	CA	407.25	343.2	60	34
**Glycolithocholic acid**	GLCA	432.25	74	60	35
**Glycodeoxycholic acid**	GDCA	448.25	74	60	35
**Glycoursodeoxycholic acid**	GUDCA	448.26	74	70	34
**Glycochenodeoxycholic acid**	GCDCA	448.26	74	48	34
**Glycocholic acid**	GCA	464.26	74	74	34
**Taurolithocholic acid**	TLCA	482.25	80	60	60
**Taurochenodeoxycholic acid**	TCDCA	498.25	80	60	60
**Taurodeoxycholic acid**	TDCA	498.25	80	60	60
**Tauroursodeoxycholic acid**	TUDCA	498.25	80	60	60
**Taurocholic acid**	TCA	514.25	80	60	64

For plasma C4 analysis, 300 μL methanol was added to 100 μL plasma samples for liquid–liquid extraction. Samples of the mixture were incubated on ice for 30 min and then centrifuged to precipitate protein at 12,000 rpm for 30 min at 4°C. The supernatant was transferred to a sample vial and analyzed in an LC–MS system (UPLC with Xevo TQS MS, Waters, Manchester, UK) in negative atmospheric pressure chemical ionization (APCI) mode with multiple reaction monitoring. The chromatographic separation was achieved on an Acquity HSS pentafluorophenyl (PFP) column (2.1 × 100 mm, particle size is 1.8 μm, Waters Corp., Milford, USA) at 25°C with mobile phase A (25% acetonitrile with 0.1% formic acid) and mobile phase B (methanol). The flow rate was set to 0.3 mL/min. The capillary voltage was 1.5 kV and the source temperature was 150°C. The desolvation gas was set at 800 L/h at a temperature of 600°C. The cone gas flow was 150 L/h. System operation and data acquisition were controlled using Mass Lynx software, and targeted metabolic data were analyzed by TargetLynx (Waters, Milford, USA).

Supplementary material – Figures

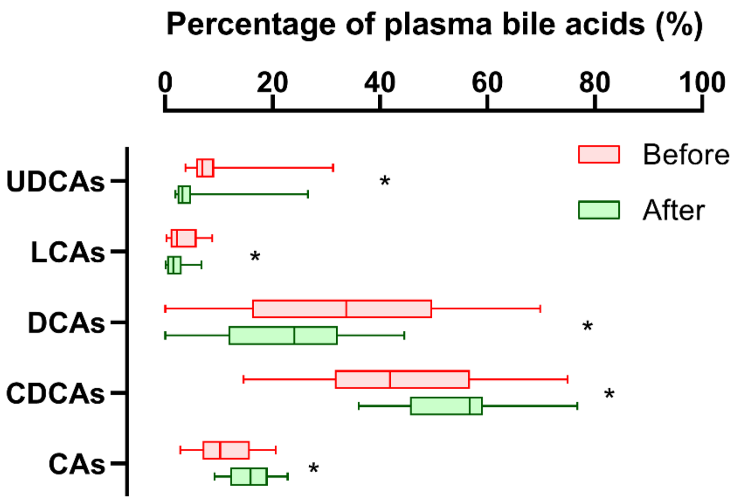

Figure S1. Changes in the percentage composition of total bile acids after YH1 treatment. * $p < 0.05$ was considered statistically significant**.**

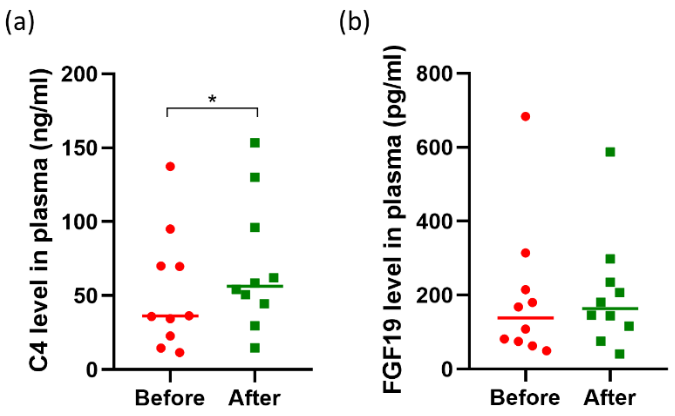

Figure S2. Plasma C4 and FGF19 levels before and after YH1 treatment.

Changes in C4 and FGF19 levels in plasma before (red circles) and after (green squares) YH1 treatment in ten patients were shown as scatter plots. The line in the graph indicated the median value. The Wilcoxon signed-rank test was used to determine whether there was a statistical difference before and after treatment (*$p < 0.05$). (a) C4 values were significantly increased after YH1 treatment.

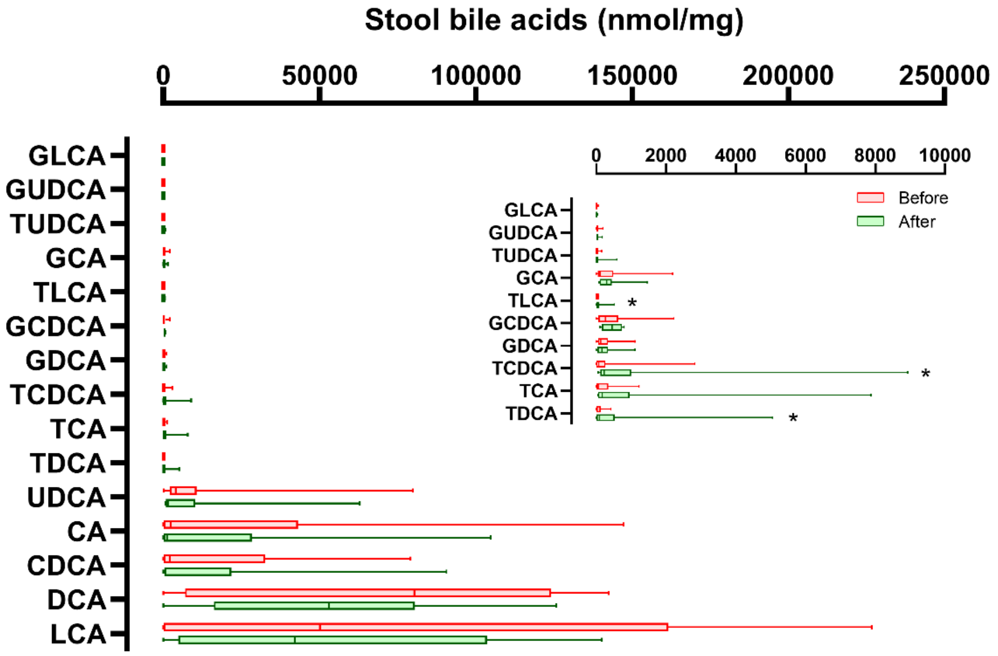

Figure S3. Stool bile acid profiles before and after YH1 treatment. * $p < 0.05$ was considered statistically significant.
